# Towards equity in organised cancer screening: the case of cervical cancer screening in France

**DOI:** 10.1186/s12905-018-0683-0

**Published:** 2018-11-26

**Authors:** Sylviane Darquy, Grégoire Moutel, Odile Jullian, Stéphanie Barré, Nathalie Duchange

**Affiliations:** 1 Univ. Bordeaux, Inserm U1219, EPICENE, Cancer et expositions environnementales, 33000 Bordeaux, France; 2 Normandie univ, UNICAEN, Inserm U1086, ANTICIPE, 14000 Caen, France; 3 Espace régional d’éthique, CHU de Caen, Normandie Université, 14000 Caen, France; 4 Institut National du Cancer, 92513 Boulogne-Billancourt, France

**Keywords:** Cervical cancer screening, Organised programme, Equity, Proportionate universalism

## Abstract

**Background:**

The French national cancer institute (INCa) conducted a series of studies to assist decision-making in view of the implementation of organised cervical cancer screening that will be launched in 2018. The programme will concern all women aged 25–65 and targeted interventions will be developed for underscreened populations. This is an evolution from an equality-based approach to a step-by-step strategy of equity aiming to tackle health cancer inequalities that are avoidable and represents unfair differences. Here we present the work of the expert-group in ethics drafted by INCa to review the ethical issues prior to the programme implementation.

**Discussion:**

We discuss the value of such a strategy and presents reflections with regard to issues of stigmatization, respect for individual freedom and autonomy. Indeed, the balance has to be found between the search for beneficence and the potential occurrence of perverse effects, which should be considered with particular attention.

**Conclusion:**

Moving toward an equity-oriented policy under a strategy of proportionate universalism faces a number of challenges, thus an overview of ethics and social sciences must be an integral part of the process.

## Background

Nationwide organised cervical cancer (CC) screening will be launched in France in the course of 2018. At present, health authorities recommend screening with the Papanicolaou test every three years for women aged 25–65 years, after two first negative tests. As it is a slow progressing disease, efficient prevention can be obtained through screening, detection and treatment of precancerous abnormalities. Cervical cancer has a worsened prognosis in case of late diagnosis. In the literature, it is widely recognised that organised CC screening is more effective than individual screening in reducing incidence and mortality [1–8]. Most European countries have implemented population based screening, or plan to do so [9], according to the European Council Recommendation [10].

In France, screening is therefore opportunistic and mainly offered through gynaecologists. There is no organised programme and no individual invitation to the target population. Even so, screening led to a decrease in the incidence of cervical cancer (standardised world standard) of 2.5% per year between 1980 and 2012 in France [11]. Over the period 2010–2012, the three-year average overall coverage rate in France was 62.3%, ranging from 41.6 to 72.5% according to geographic location [12], but it has been shown that among the 38% of women who had no Pap-smear, those in vulnerable situations accounted for a significant proportion [13].

The objective of organised CC screening is to reduce the incidence and number of deaths by increasing the coverage rate of screening in the target population. The French national cancer institute (INCa) conducted a series of studies to assist decision-making in view of its implementation [14–16]. These studies first evaluated the cost-effectiveness of different screening strategies and then quantified non-participating women, leading to an accurate assessment of the means that the forthcoming organised programme should deploy. Pilot programmes were established before generalisation across the country and have shown that this may improve the uptake of cervical cancer screening [12]. All together, the results clearly confirm the importance of the generalization of CC screening and the need of targeted actions in order to better reach non-participant populations.

The proposed organisational framework is a programme open to all women aged 25–65, with targeted interventions for identified under screened populations. This strategy aims to address socio-economic inequalities found in the existing organised programmes for cancer screening, in line with the established priorities of the national French Cancer Plan 2014–2019.

Taking into account social inequalities in health is essential in the implementation of public health programmes to avoid loss of opportunity. A universal approach does not take into account the potential barriers that limit access for some populations. A “graduated approach” aims to reduce differences between socio-demographic groups to the extent that complementary actions are implemented that benefit the populations that were excluded from them.

For this reason, for the first time in France, the design of organised screening is thus shifting from a universal logic, based on equality, to a strategy of proportionate universalism, a concept according to which health actions are universal with a scale and intensity that are proportionate to the level of disadvantage [17]. As highlighted by Benach et al. [18], there is a distinction between universal policies that include additional targeting of deprived populations, and proportionate universalism that increases benefits along the social gradient. This is an evolution from an equality-based approach to a step-by-step strategy of equity aiming to tackle health cancer inequalities that are avoidable and represent unfair differences. The recurrent observation of lower participation among deprived populations and minority groups revealing inequalities in access. This situation motivates the setting of targeted interventions that are currently gaining ground in different European countries (for a review see [19]). Different forms of universalism and targeting are distinguished and different meanings are attributed to these concepts [20]. Therefore, it is important to clarify the design of the program and interventions especially since each public health programme has its own particularities and the assessment of the benefit/risk balance is a decisive factor in justifying the proposed actions.

In practice, the move towards an equity-oriented policy within a strategy of proportionate universalism faces a number of challenges and ethical considerations. This approach to screening requires the use of social, behavioural or subgroup criteria, with the risk of interfering with privacy and of stigmatising. Ethical reflection in the management of organised cancer screening programs has been introduced since 2009 with the setting of a dedicated group of experts coordinated by INCa. The objective is to combine expertise from ethics and social sciences with that of epidemiologists, public health policy authorities and practitioners involved in the coordination and operation of the screening programmes. In this paper, we present the conclusions of the group who addressed the issues at stake and analysed the proposed strategy prior to the implementation of the organised CC screening programme [21].

## Discussion

### A public health programme that aims at equity

The efficacy of CC screening in reducing incidence and mortality is well recognized. Contrarily to other screening programmes where the balance benefit/risk is debated, It is described as an ‘exemplar population screening programme’ [22] justifying the commitment of policy-makers to reduce the loss of opportunity of women identified as non-participant to individual screening [13].

Inequalities in health refer to the relationship between health and social category. There is a close correlation between socioeconomic status and health: this is called the “social gradient of health” [23]. Actions designed to fight social inequalities in health are intended to develop an integrated approach, in terms of health education, prevention, screening and access to care [24].

Inequalities in cancer occur at each stage of the life and health trajectory (exposure to risk factors, access to prevention, participation in screening, quality of care pathways, post-cancer conditions) [25]. Inequalities reflect a cumulative “loss of opportunity” face to the cancer. A study in France has demonstrated that women’s low social status is a factor in the occurrence of cervical cancer [13].

It is therefore essential to consider this reality when implementing public health programmes. The universal approach is designed for the entire population concerned, without taking into account the potential barriers that limit access for some populations. Policies are concerned with creating equal conditions for health and reducing health disparities between socio-economic groups. This notion of health equity is part of a theory of social justice aimed at the implementation of favourable health conditions for all [26, 27]. From this point of view, the choice was made to organise the CC screening programme in accordance with the principle of proportional universalism [13, 28]. This approach aims at equity in order to limit the loss of opportunity for those categories of women identified as non-participants [24].

### French organised cervical cancer screening strategy

The programme therefore consists of a global strategy intended for all women aged 25–65, along with complementary actions targeted towards the groups of non-participating women. The INCa medico-economic studies [14, 15] have evaluated various possible CC screening generalisation scenarios, their prioritisation in terms of participation gains, their ability to reduce inequalities and their financial impact. On this basis, the different targeted actions will be gradually deployed and evaluated [16].

Targeted strategies are planned according to whether or not women have been identified as participants in opportunistic screening in recent years. The identification of subgroups of non-participating women will make it possible to adapt information and awareness raising as well as to develop specific experimentations (Table 1). For women over 50 years of age, specific information is proposed because many of these women wrongly consider that the risk diminishes after age 50 [29]. For women unaware of their risk, information will be reinforced because these populations are under-informed about risk [30]. For vulnerable populations, it is planned to send medical and para-medical personnel to offer screening directly to populations. Lastly, for the category of high-risk women, specific information will be developed, as these women are often unfamiliar with the over-risk category [31].

**Table 1:** Proposed organisation of the French cancer cervical screening programme

All women 25–65 years of age		
Global Strategy	1. Collective information campaigns and individual invitation 2. Training and diversification of health professionals (physicians, midwives, nurses) 3. Diffusion of recommendations to professionals, in particular for the follow-up of pregnant women (gynaecologists, general practitioners, midwives) 4. Practice harmonisation (standard, recommendations) 5. Data collection improvement 6. Evaluation	
Targeted Strategies	Criteria: No Pap-smear recorded for 3 years or more	
	Women over 50 years of age (age at which participation declines)	→ Collective awareness-raising for both women and health professionals → Individual invitation and information
	Women unaware of their risks: precarious or homosexual women	→ Collective awareness-raising → Targeted information relayed by associations
	Vulnerable populations/ populations remote from the health system (prostitutes, Roma, migrants...)	→ Implementation of targeted screening actions on the ground → Intervention of local and relevant associations → Targeted information → Action evaluation
	Women at increased risk of cervical cancer (HIV, immunosuppression, diethylstilbestrol)	→ Awareness-raising for both women and health professionals → Information relayed by associations

The ethical issue is the identification of target groups and the actions that will be offered to them. It will be necessary to define priorities for action in the light of the results of the experiments. Choices regarding the allocation of available resources will have to be made and justified. This decision-making process requires transparency in the criteria that will guide choices.

### Address the risk of stigmatisation in targeted interventions

Subgroups in which a significant proportion of specific health problems are observed are often subject to pre-existing social devaluation. Public health actions aimed at groups with characteristics negatively perceived by society raise ethical questions related to the risk of stigmatisation [32].

Health-related stigma is typically characterised by social disqualification of individuals and populations who are identified with particular health problems and behaviours related to specific socio-cultural and economic conditions.

Serious ethical concerns arise through the targeting of subgroups of populations according to certain characteristics. For migrant populations for example, the fear of stigmatisation could prevent the implementation of actions towards them. When addressing this population in the name of the principle of beneficence, our recommendation is to recall that the principle of autonomy must be respected and that screening is not mandatory. To this end, health professionals must act in accordance with consent and confidentiality.

Similarly, for precarious or homosexual populations of women, information could be reinforce through associations or communities themselves. Meanwhile, training of health professionals has to be improved.

Targeted actions in public health therefore require the ethical relevance of the criteria and methods of action adopted to be called into question [33]. This is referred to as positive discrimination in the sense that it facilitates access to care and tends to reduce health inequalities.

In France, these questions have emerged in several public health plans. For example, it was proposed to suppress the obligatory character of vaccination against tuberculosis and instead to target it to populations at risk, mostly disadvantaged people. Targeting is intended to benefit these precarious populations at risk but involves ethical issues that go beyond medical issues alone, as it implies specifying the characteristics of a population. Tuberculosis is indeed a disease whose occurrence is encouraged by precarious conditions and migration from endemic countries, presenting the risk of revealing a social context at the same time as the disease. The French national ethics committee has been challenged to address these issues [34]. The Committee agreed on the principle of abolition of compulsory vaccination but produced recommendations as regards targeting these populations, in particular respect for the fundamental freedom of the individuals, including the right to refuse. It also recommended deploying appropriate information procedures. The contribution of ethical reflection consists in clearly identifying the values at stake and, above all, in establishing those which should guide the decision, in order to assess the reasonableness of public health activities that may present risks of stigmatisation towards certain subgroups of the population.

Thus, the risks must be taken into account and weighed against the expected benefits. Actions with potential risk of stigmatization have to be justified by evaluating their effective contribution in promoting health benefits for the people concerned. On another hand, if the risk of stigmatisation is proven, this may justify not implementing the action in question. A balance has to be found between the search for beneficence and the degree of potential perverse effects; actions to reduce those have to be sought.

### Respect for individual freedom and privacy

There are two approaches in public health, one is the obligation, as is the case for vaccination, which has recently been reinforced in France [35], and the other is on the principle of voluntary participation. Participation in cancer screening is based on respect for autonomy, meaning that people are asked to decide whether to participate in screening. As discussed by Parker et al. [36], this is not a simple task, particularly when the levels of benefits and harm are close and prevents experts from making strong recommendations. As the efficiency of a programme relies on the level of participation, it is important that its promotion is balanced with the provision of an information that includes benefits but also potential risks and harms so that people can make an informed choice [37].

Making every effort to increase participation is a laudable objective when 1) there is a favourable risk-benefit balance, as in CC screening when the detection of precancerous lesions has an impact on the incidence of cancer, and 2) the underlying issue is to fight against social inequalities. However, as low participation may call into question the efficiency of a public health action, vigilance is required as beneficence is not an absolute that could a priori justify all the actions that may be decided upon. Thus, it is essential to achieve the desired health objectives while minimising the degree of intrusion into the private sphere, and the benefit it generates must be justified [38, 39]. This may also lead to vigilance on how targeted actions may potentially conflict with respect for individual freedom and privacy according to the principle of autonomy. This calls for an evaluation of the degree of paternalism that can be justified and acceptable in the quest for the common good that is health [40–43].

One way of setting limits on the beneficence imperative and interventionism would be to apply Feinberg’s proposed distinction between weak (or soft) and strong (or hard) paternalism [44]. In a weak paternalism, public authorities seek to elicit the informed consent of people rather than compel them to act in what appears to be their best interests. Weak paternalism does not preclude the concept of promoting autonomy, therefore, as it gives priority to the informed exercise of the ability to make a choice [45, 46]. The CC screening programme is designed with a low paternalism perspective and must ensure the autonomy of the populations concerned, particularly those identified as vulnerable.

The target actions aim at reinforcing information and awareness raising on access to screening within the framework of respect for autonomy and freedom to participate.

## Conclusion

Organised cervical cancer screening will be launched in France in 2018 based on proportionate universalism, a strategy used for the first time for organised cancer screening in the country. The aim is to provide equal access to the test to the whole target population and to implement complementary actions directed toward non-participant women previously identified. This will serve take into account certain needs. The programme, together with specific actions, will be implemented gradually with regular monitoring to assess their relevance. An overview of ethics and social sciences are and will be an integral part of the process, as the balance between benefits and risks has to be evaluated on an ongoing basis. The establishment of a proportionate universalism must take into account the psychosocial reasons for social inequalities in health and promote actions such as combating social isolation, restoring the feeling of being able to exercise control over one’s life, and supporting individual and community empowerment in order to facilitate citizen reintegration [47].

## Abbreviations

CC: Cervical cancer
INCa: French national cancer institute
